# A Comparison of Self-Reports and Unobtrusive Multimodal Measures of Stress in a Realistic Simulated Task Environment

**DOI:** 10.3390/s26154962

**Published:** 2026-08-05

**Authors:** Nidhal Mazza, Kexin Feng, Benjamin D. Schulte, Theodora Chaspari, Winfred Arthur

**Affiliations:** 1 Department of Psychological and Brain Sciences, Texas A&M University, College Station, TX 77843, USA; bschu5@tamu.edu; 2 Department of Computer Science and Engineering, Texas A&M University, College Station, TX 77843, USA; kexin@tamu.edu; 3 Institute of Cognitive Science, Department of Computer Science, University of Colorado Boulder, Boulder, CO 80309, USA; theodora.chaspari@colorado.edu

**Keywords:** self-report, unobtrusive measurement, stress, physiology, computer interaction, facial expression, simulated task environment

## Abstract

Although a popular measurement method in different fields, self-report measures have multiple shortcomings that stem from their very inherent reliance on the participant’s subjective judgment or recall. Given these limitations, in the domain of stress research, unobtrusive measures have been proposed as alternatives. Using a sample of 78 university students who participated in a disaster response simulation in a synthetic task environment, the present study sought to examine whether unobtrusive measures of stress (i.e., physiological, computer interaction, and facial data) can serve as valid substitutes for self-report measures (i.e., state anxiety, task load, and momentary stress). To that end, the study compared the extent to which features from these different modalities accurately (1) classified participants into the stress condition to which they were assigned as well as (2) predicted their performance on a complex synthetic task. The results indicated that the unobtrusive measures individually predicted task performance better than self-report measures, whereas self-report measures predicted the stress condition to which participants were randomly assigned (i.e., *no-stress*, *low-stress*, and *high-stress*) better. Therefore, in spite of their limitations, self-report measures cannot be systematically substituted by unobtrusive measures; this may depend on the outcome of interest. Future research is needed to examine the generalizability of the findings and explore the criterion-related validity of other modalities of stress.

## 1. Introduction

Self-report measures are one of the most popular if not the most popular method of measurement in the behavioral, social, and organizational sciences [1,2]. Their ubiquity is justified by their several advantages. First, self-report measures are relatively easy to develop compared to other methods and can be designed to assess a wide range of constructs. Their administration also requires minimal expertise and resources, and they can be administered simultaneously to large groups of participants [3]. Finally, self-report measures are unique in their ability to capture subjective and/or covert information, which can be difficult if not impossible to access via other methods (e.g., self-perceptions; [3,4]).

However, self-report measures have multiple shortcomings that stem from this very inherent reliance on the participant’s subjective judgement or recall. Indeed, they may be susceptible to multiple response biases such as careless responding, which takes place when participants respond with little to no attention to the item content [5], or socially desirable responding, which refers to participants’ tendency to misrepresent themselves generally in a favorable manner [6]. A final limitation of self-reports is that they can be intrusive to real-time tasks if completed simultaneously [7]. That is, asking participants to provide self-reports while performing a task is likely to cause disruption to the performance of the task.

The limitations of self-report measures are vividly highlighted in the domain of stress research, where unobtrusive measures have been proposed as alternatives that circumvent the noted limitations. Indeed, obtaining unobtrusive measures of stress is a research direction that has seen a burgeoning increase in popularity in the fields of psychology, medicine, and engineering. Due to the increasing physical, cognitive, and emotional demands of the modern world, stress is becoming a global epidemic with a 29% prevalence worldwide [8]. Stress is generally defined as an individual’s physiological and psychological response to a challenge or threat from the environment that exceeds their coping capacity [9]. Based on its duration, it can be categorized as chronic or acute. Chronic stress arises from prolonged exposure and has been linked to negative health outcomes. In contrast, acute stress results from short-term events (e.g., cognitive demands, interpersonal conflicts) but can potentially lead to chronic stress if the physiological responses remain unresolved over time [10]. Acute stress is typically assessed via self-reports, which are limited by subjectivity, careless responding, socially desirable responding, and low temporal resolution. Being both objective and passive, unobtrusive measures of stress have the potential to address some of these limitations, while also enabling continuous stress assessment and subsequently, detection. This, in turn, potentially enables the design of timely interventions to reduce the negative effects of stress by administering them at opportune moments [11]. Common types of unobtrusive measures that have been used for detecting stress include computer interaction measures which capture the interaction between the user and the computer via a mouse and keyboard clicks, facial measures which capture facial expressions via a camera, and physiological measures which capture the body’s internal responses to stressor stimuli.

Stress can affect the *autonomic nervous system (ANS)*, also known as the involuntary nervous system since its functions tend to be unconscious without voluntary control [12]. For example, the sympathetic nervous system (SNS) is a component of the ANS that is responsible for the body’s “fight-or-flight” response to dangerous or stressful situations. Therefore, as it pertains to physiological measures, the activation of the SNS due to a stressor event can cause increased activity of the sweat glands that is manifested in changes in electrodermal activity (EDA) [13], pupil dilation [14], the narrowing of blood vessels that leads to a decrease in the blood volume pulse (BVP) [15], and an increase in heart rate [16]. On the other hand, *the parasympathetic nervous system (PNS)* is responsible for the “rest and digest” function of the body and indicates a state of recovery from the stressful stimuli manifested as a decrease in sweat activity, slower heart rate, and constricted pupils [17]. One’s ability to effectively modulate their heart response can result in increased heart rate variability [18,19], which can be quantified using time- and frequency-domain features of the electrocardiogram (ECG) signal [20].

A stressful event can also impact facial and head movement. For instance, increased stress can potentially cause more frequent and more rapid head movement, asymmetric lip deformations, and reduced frequency of mouth opening [21,22]. Stress may further lead to increased tension in the speech musculature and compromise one’s ability to control one’s vocal expressions, thus impacting speech output and resulting in changes in vocal measures [23,24].

Studies have explored features from multiple modalities for stress detection, primarily employing supervised machine learning algorithms. A comprehensive review of the features and models used for automatic stress detection can be found in [22]. Physiological measures generally yield moderate classification performance in automatic stress detection. For example, in a study where stress was induced using the Montreal Imaging Stress Task, EDA features achieved a binary classification accuracy between 60.5% and 72.3% for classifying between the absence and presence of self-reported stress [25]. Similarly, analyzing data from 18 participants who completed the Trier Social Stress Test resulted in an 88% binary classification accuracy using EDA features and heart rate features [26]. Despite these positive results from simulated stress in controlled settings, detecting stress in realistic task settings and environments remains challenging due to the weaker links between daily life stressors and physiological responses compared to behavioral responses [27]. For instance, physiological measures alone achieved a 64% accuracy in binary classification accuracy when estimating stress from naturalistic office-related work tasks, while computer interaction features produced a similar accuracy of 65% for the same task [28]. Some researchers have also developed stress detection systems based on facial action units. For instance, a support vector machine (SVM) classifier utilizing facial action units to identify stress achieved an accuracy of 77% [29]. More recent studies, such as [30], have identified common facial patterns associated with stress, offering a more interpretable method for stress detection. Other studies have combined facial action unit measures with features that capture head orientation, overall facial movements, and emotion, resulting in 75% binary classification accuracies [28].

The present study examined stress in the context of performance on a task-type known as synthetic task environments (STEs). STEs, which are extensively used in the individual and team performance literatures, are complex performance tasks that simulate and model the cognitive, information-processing, psychological, and in team contexts, the social processes and demands that are present in operational environments [31,32]. STEs permit the examination of complex performance processes in controlled experiments in a manner that is typically not feasible in actual operational settings. Thus, STEs, which include but are not limited to computer-based simulators and desktop trainers, can be configured to simulate a wide range of conditions and events, and also allow for the collection of detailed performance data. As reflected in [32], computer-based simulations of this sort have clear research informative value and utility because “STEs provide a valuable compromise between the complexity of the real world, which is important and critical for establishing externally valid results, and experimental control, which is necessary to establish internally valid results.” [33]. These advantages explain their widespread use in training and complex skill acquisition research [32].

Given the limitations of self-report measures and the increasing popularity of unobtrusive measures, the goal of the present study is to examine the extent to which unobtrusive measures of stress can serve as valid substitutes for self-report measures of stress. Specifically, using a concurrent design, we investigate the criterion-related validity of three types of unobtrusive measures of stress (i.e., physiological, computer interaction, and facial data), that is, the extent to which they can predict two outcomes: (1) the stress condition to which participants are assigned (i.e., *no-stress*, *low-stress*, and *high-stress*) and (2) performance on a complex synthetic task, better than self-report state measures (i.e., state anxiety, task load, and momentary stress). The use of these two different outcomes allows for a more comprehensive comparative evaluation of the efficacy of unobtrusive measures as valid alternatives for self-report measures of stress. To address this question, we analyzed data collected in a synthetic task environment called *Crisis in the Kodiak: Oilrig Search and Rescue* [31,34], which is a prototypical example of the types of complex synthetic task environments used in lab-based training and performance research.

This study contributes to the literature in several ways. First, the majority of studies on automated stress detection have explored different stress operationalizations individually [22], leaving the potential of comparing and contrasting among the types of stress outcomes largely underexplored. To address this gap, the present study uses two complementary stress outcomes (i.e., the experimentally assigned stress condition and task performance) to assess the comparative criterion-related validity of self-reports and different types of unobtrusive measures (i.e., physiological, computer interaction, and facial data). The use of these two different outcomes allows for a more comprehensive comparative evaluation of the utility of the different modalities as measures of stress. Second, unlike prior studies that typically evaluate stress detection at a single time point [25,26], the data are collected at multiple time points (i.e., 4 trials) which allows for an examination of the temporal stability of their criterion-related validity. Third, data analyzed in this study were collected via a synthetic task environment (STE), which is a noteworthy contribution to the extant literature because most studies in this field have focused on simulated stress during tasks that do not realistically simulate real-life stress conditions (e.g., Stroop Test, Mental Arithmetic Task) [21,25,26], and there are mixed results regarding the feasibility of automated stress detection from real-world data.

## 2. Materials and Methods

### 2.1. Participants

The data used in the present study are part of a larger data collection effort. The final study sample consisted of 78 students recruited via the bulk email service at Texas A&M University (College Station, TX, USA). Eligibility criteria were that participants must be between 18 and 30 years of age, fluent in English, possess previous experience working with a computer, have no diagnosis of a neurological or psychological condition that affects their ability to focus, and possess no previous experience with the performance task. Additionally, because the experimental manipulation included aural stimuli, participants were required to obtain a passing score on a hearing ability test (www.hearingtest.online, last accessed on 1 April 2021).

Participants were compensated one $50 Amazon gift card for completion of the study. Participants were also made aware that one $20 Amazon gift card would be awarded to the participant who obtained the highest task performance score in their particular study session. The performance reward was meant to motivate participants to perform at their maximum ability on the performance task. A study session consisted of two to four participants. On average, participants were 21.83 years of age (*SD* = 3.46). Table 1 presents additional demographic information.

Self-report measures of stress (Section 2.3), unobtrusive measures of stress, which consisted of physiological, computer interaction, and facial measures (Section 2.4), and performance measures (Section 2.2) were obtained from participants. Table 2 presents the list of self-report and performance variables and their descriptive statistics. The lists of the unobtrusive measures are presented in Appendix A (Table A1, Table A2 and Table A3).

### 2.2. Performance

**Figure 1:**
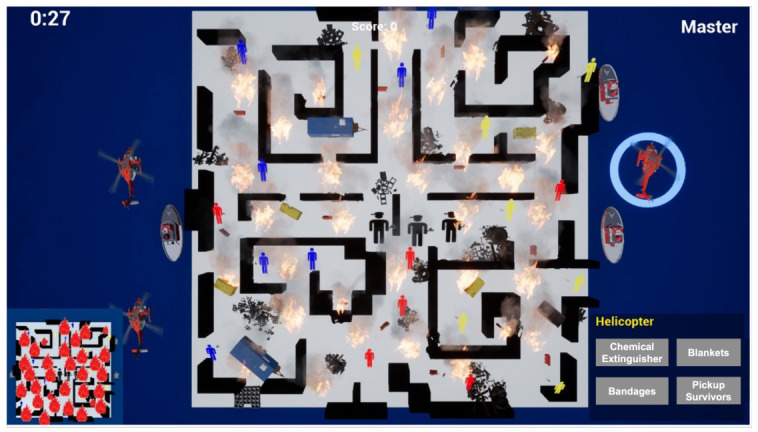
Screen capture of *Crisis in the Kodiak: Oilrig Search and Rescue*.

**Figure 2:**
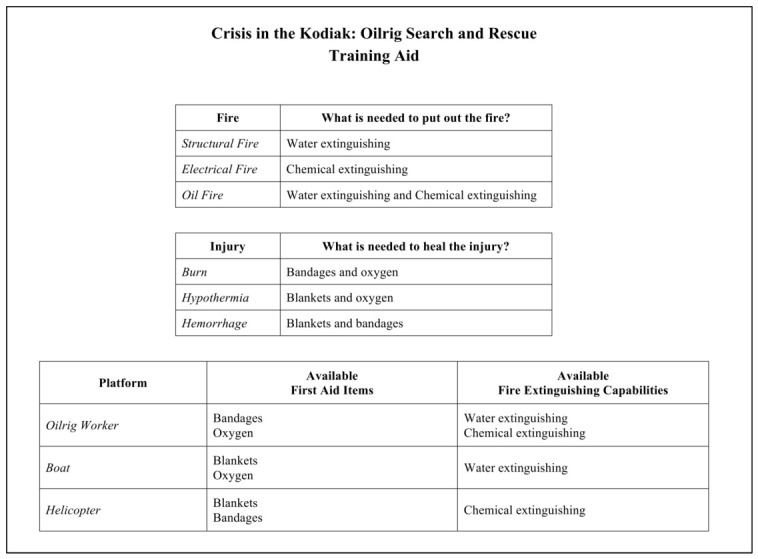
Onscreen task/training aid.

The performance task was *Crisis in the Kodiak: Oilrig Search and Rescue* [31,34,35]. *Crisis* is a dynamic, computer-based, point-and-click synthetic task environment. It was designed to not require any previous video game experience. Participants operated the simulator as individuals through a command-and-control interface using a desktop computer with two monitors and a mouse. A full and detailed description of this training and performance task is reported in [34]. A screen capture of the *Crisis* task screen is presented in Figure 1 and the task/training aid—which presented a synopsis of major task-related information pertaining to the types of fires, injuries, and platforms—that was displayed on the second monitor, is presented in Figure 2.

*Crisis* is a disaster response simulator in which a participant operates three roles (corresponding to three platforms)—oil rig workers, boat captains, and helicopter aviators—and is tasked with responding to an offshore oil rig explosion. Each role consists of three platforms (i.e., boat captain has 3 boats, helicopter aviator has 3 helicopters, and oil rig worker has 3 oil rig workers). Thus, each participant individually operates nine platforms. Additionally, each role varies in its capabilities on six dimensions: (1) range of vision, (2) speed of movement, (3) type of movement (land [oil rig worker], air [helicopter operator], and sea [boat operator]), (4) type of capabilities (different methods for extinguishing fires and different resources for healing survivors), (5) range of these capabilities, and (6) the ability to pick up survivors.

Within the context of the primary task objectives of healing and rescuing survivors, and extinguishing fires (to be able to shut off leaking oil valves, the source of the fires), as indicated in Figure 2, there were three types of fires—structural, electrical, and oil—each requiring a different agent to extinguish it (e.g., chemicals for electrical fires). Likewise, there were also three types of injuries to be healed—burns, hypothermia, and hemorrhages—again, each requiring different resources to do so (e.g., bandages and oxygen to treat burns). Finally, as previously noted, and also indicated in Figure 2, the three different platforms (oil rig worker, boat, and helicopter) had different capabilities and resources to treat injuries and extinguish fires. Therefore, for instance, boats can treat only burns and hypothermia because they carry blankets and oxygen, and can extinguish only structural fires because they carry water. As another example, because of differences in their type of movement (i.e., land, air, or sea), neither helicopters nor boats can shut off valves, whereas oil rig workers can. Thus, in summary, during each performance trial, participants interactively operated nine platforms (three for each of the three roles), integratively using their capabilities to achieve the two goals of shutting off leaking oil valves (to *extinguish fires* to accomplish this) and *healing and rescuing survivors*.

Participants earned 10 points per healed survivor, 10 points per rescued survivor (survivors had to be healed before they could be rescued), and 50 points per oil valve shut off. Each performance trial consisted of 20 survivors and four oil valves. Therefore, the maximum possible score was 600 points per trial. The method used to determine performance scores was explained to participants during training, and scores were available during mission performance. As explained in more detail in Section 2.5, performance trials had different durations across the stress conditions. Specifically, the *no-stress*, *low-stress*, and *high-stress* conditions consisted of 10-min, 8-min, and 6-min trials, respectively. To make performance scores comparable across conditions in the analyses, performance scores of trials that lasted 6 or 8 min were scaled up to the equivalent scores for a 10-min trial using the following formula: scaled score of the trial = original score of the trial × 10/duration of the trial. Correlations between task performance scores in consecutive trials post the first trial (i.e., test-retest reliability) were high, that is, 0.73 between Trial 2 and 3, and 0.66 between Trial 3 and 4.

### 2.3. Self-Report Measures of Stress

Stress was measured using three types of self-reported measures: state anxiety, momentary stress, and task load.

#### 2.3.1. State Anxiety

State anxiety was operationalized as mean scores on the 20-item State-Trait Anxiety Inventory (STAI) [36] Form Y-1. An example item is “*I am tense.*” Participants responded to each item on a 4-point scale (1 = *not at all*; 4 = *very much so*) according to how they “*feel right now, that is, at this moment*.” The measure was administered after each trial. The levels of internal consistency (i.e., the extent to which the items display homogeneity) for state anxiety scores were acceptable for each administration (αs ≥ 0.90).

#### 2.3.2. Momentary Stress

Stress experienced during the performance task (i.e., momentary stress) was operationalized as scores on a single item (i.e., “*At present I feel…*”). Participants responded to the item on a 7-point scale (1 = *very stressed*; 7 = *very relaxed*). The item was presented in a pop-up window three times during each performance trial on a fixed interval schedule, specifically, every third interval of the trial length. For instance, in a 6-min trial, the item was administered after 2 min, 4 min, and immediately before the trial ended. Scores for each trial were computed as the mean of the three administrations, reversed such that higher scores indicated higher levels of momentary stress. Test-retest reliability estimates (i.e., correlations between different administrations in a given trial) were high, ranging from 0.62 to 0.73 in Trial 1, 0.72 to 0.78 in Trial 2, 0.61 to 0.77 in Trial 3, and 0.79 to 0.85 in Trial 4.

#### 2.3.3. Task Load

Task load was operationalized as mean scores on four items from the NASA-Task Load Index (NASA-TLX) [37]. The items pertained to participants’ perceptions of the effort, frustration, as well as the mental and temporal demands associated with the performance task. An example item is “*How mentally demanding was the task?*” Participants completed the items using a slider scale from 0 to 100. The measure was administered after each trial. Task load scores showed somewhat low levels of internal consistency (i.e., the extent to which the items display homogeneity) for each administration (0.61≤αs ≤0.73).

### 2.4. Unobtrusive Measures of Stress

In addition to the self-report measures, stress was also measured during task performance using three types of unobtrusive measures: physiological, computer interaction, and facial.

#### 2.4.1. Physiological Measures

Physiological stress was measured using Actiwave Cardio devices (CamNtech Inc., Boerne, TX, USA) [38] attached to participants’ chests and Empatica E4 (Empatica Inc., Cambridge, MA, USA) sensors [39] attached to participants’ wrists. These devices measured physiological signals. Table A1 presents the list and description of the physiological measures of stress extracted from each physiological signal and its respective device.

Following their extraction from the Empatica E4 and Actiwave devices, and prior to the analyses, the sensor data were processed and curated. First, any sensor data extracted outside the duration of the performance trials were excluded before further processing. The Empatica E4 device measured participants’ BVP, EDA, and wrist Acceleration (ACC). For the EDA signal, it was first smoothed using a low-pass filter and then normalized into a standard normal distribution. Then, the skin conductance level (SCL) was calculated as the mean of the smoothed EDA signal. The amplitude and frequency of the skin conductance response (SCRAmp and SCRFreq, respectively) were also derived from the smoothed EDA signal using the Matlab-based Ledalab software (http://www.ledalab.de/, last accessed on 3 February 2024). SCL, SCRAmp, and SCRFreq were extracted within a 10 s analysis window and subsequently further averaged for each trial. The BVP signal was processed by calculating the mean interbeat interval (IBI), mean heart rate (HR_mean.1), and mean level of BVP (BVP_mean) for each 10 s analysis window. The energy of the ACC signal (i.e., root of the average square value of the signal) was also computed using the same analysis window, which serves as a measure of overall movement (ACC_enrg). The measures derived every 10 s from the BVP signal were further averaged across each trial of the study. This process yielded a total of seven features from the Empatica E4 device for each trial.

The Actiwave device measured participants’ ECG, and the collected signal was first denoised using the BioSPPy Python package (https://biosppy.readthedocs.io/en/latest/, last accessed on 3 February 2024). The hrvanalysis Python package (https://pypi.org/project/hrv-analysis/, last accessed on 3 February 2024) was used to derive time-domain metrics based on the N-N intervals (NNI). This includes 5 fundamental descriptors of the N-N intervals: mean (NNI_mean), standard deviation (NNI_sd), range (NNI_range), median (NNI_median), and coefficient of variation (NNI_cv). To capture potential discontinuities in the NNI, 7 additional features were extracted, which consist of the number of successive NNI pairs differing by more than 50 or 20 ms (NNI_50 and NNI_20, respectively), the percentage of successive NNI pairs differing by more than 50 or 20 ms (pNNI_50 and pNNI_20, respectively), as well as the standard deviation (NNIDiff_sd), root mean square (NNIDiff_rms), and coefficient of variation (NNIDiff_cv) of successive differences between NNIs. Four heart-rate descriptors were also extracted using the NNI, which are the minimum, maximum, mean, and standard deviation of heart rate (HR_mean.2, HR_max, HR_min, and HR_sd). In addition to the time-domain measures, 7 frequency-domain measures, including the very low, low, and high-frequency power of the ECG (VLF, LF, and HF, respectively), the normalized low and high-frequency power of the ECG (LFnu, and HFnu, respectively), the ratio of low-frequency to high-frequency power of the ECG (LF_HF_ratio), and the total spectral power (Total_power) were extracted. The Actiwave device also has an integrated heart rate sensor, allowing for the computation of the mean HR (mean_HR.3). All the above measures were extracted for each 10 s analysis window and were subsequently averaged for each trial. Following data processing and curation, a total of 31 high-level features (7 from Empatica E4 and 24 from Actiwave) were obtained for every trial.

During the data processing steps, several 10-s analysis windows that were invalid likely due to inappropriate sensor usage were identified. Consequently, windows with a mean heart rate below 60 or above 100 were removed because 60–100 is the normal range for resting heart rate suggested by Mayo Clinic [40]. Furthermore, windows with a skin conductance level below 0.05, which is a threshold for abnormal values of SCL [13] were also removed.

#### 2.4.2. Computer Interaction Measures

Because the performance task required the use of a computer mouse, computer interactions were measured using mouse clicks. A Python script which recorded all mouse input information was installed on the computers that ran the performance task. Table A2 reports the list and description of each mouse movement and click used as a stress indicator.

The mouse data were processed and curated prior to the analyses. First, any data extracted outside the duration of the performance trials were excluded before further processing. A total of 10 mouse features were created. The total number of mouse clicks per trial was first computed, then the number of clicks per min (*Clicks*) was calculated by dividing the total number of clicks by the duration of the trial. Next, extreme values of the coordinates from all clicks were identified to determine the minimum and maximum values for both the X and Y axes (*X-axis.min, X-axis.max, Y-axis.min, Y-axis.max*). Additionally, the number of directional changes along the horizontal (X-axis) and vertical (Y-axis) was assessed by examining the click coordinates and adjusting these data to trial length (*Reversals_X-axis, Reversals_Y-axis*). The time difference between the trial’s start timestamp and the timestamp of the first click was computed as the initiation time (*InitiationTime*). To derive the click interval features, the average time differences across each trial between successive clicks were calculated based on their timestamps (*ClickInterval.Speed*). Successive time differences in terms of the previous variable were computed to obtain a proxy of mouse click acceleration per trial (*ClickInterval.Acc*).

#### 2.4.3. Facial Measures

Facial indicators were measured using facial action units (AUs) from the Facial Action Coding System [41]. AUs represent anatomically distinct facial muscle movements. In the present study, 41 AUs were used as facial indicators of stress. Table A3 presents the list and description of each AU.

The AU data were processed and curated prior to the analyses. Participants’ faces were videotaped using a webcam that was attached to the screen displaying the performance task. Any video data collected outside the duration of the performance trials were excluded before further processing. Two pairs of annotators (i.e., undergraduate research assistants) reviewed the entire duration of each participant’s videotaped study session and recorded the timestamps (i.e., start times and end times) for each performance trial. The annotators also recorded the timestamps during the performance trials at which the participants failed to attend to the performance task (e.g., looked away from the computer screen, asked the study proctor a question). The pairs of annotators then met to review their coding, discuss any disagreements, and reach consensus on the correct timestamps. The non-performance task and off-task behaviors video segments were then removed before further processing. The remaining video segments from the actual trials were processed using OpenGraphAU (https://github.com/lingjivoo/OpenGraphAU, last accessed on 3 October 2023) to extract AU data for every 1 s. AU scores pertained to intensity and occurrence, which were computed as the mean and standard deviation of AU occurrence, the mean and standard deviation of AU intensity, and the number of times that an AU was activated, respectively. This resulted in a total of 205 AU features that were averaged across the duration of each trial.

### 2.5. Design and Procedure

The study used a 4 (Time [within-subject]: Trials 1, 2, 3 and 4) × 3 (Stress [between-subject]: no stress vs. low stress vs. high stress) mixed experimental design. Participants were randomly assigned to one of three stress conditions, *no stress* (*n* = 26), *low stress* (*n* = 25), or *high stress* (*n* = 27) and completed four trials in total. Participants across all three conditions completed the same baseline performance trial that was 10 min long (i.e., Trial 1). However, stress was manipulated in subsequent trials (i.e., Trials 2, 3, and 4) using time constraints and distracting audio. Therefore, the *no-stress* condition consisted of 10-min performance trials with no noise. The *low-stress* condition consisted of 8-min trials with an unintelligible conversation about iron working. And the *high-stress* condition consisted of 6-min trials with an intelligible conversation about boat piloting. The unintelligible and intelligible audio files were chosen based on the speech intelligibility index [42], a measure ranging between 0 (low intelligibility) and 1 (high intelligibility), which is also associated with SCL (the mean speech intelligibility indices of the unintelligible and intelligible conversations differed in the expected direction (0.211 dB and 0.268 dB, respectively). Additionally, [43,44] found that the mean difference in SCL between intelligible and silence conditions (*d* = 0.27) was greater than that between unintelligible and silence conditions (*d* = 0.21), suggesting that the intelligible conversation would elicit more stress during the performance task than the unintelligible conversation.). Additionally, the audio files were reviewed to ensure that they corresponded to perceptually low- and high-intelligibility conversations. During each performance trial, one computer monitor displayed the performance task (see Figure 1) while the other monitor displayed a task/training aid regarding *Crisis* (see Figure 2). Appendix B presents the detailed study procedure (Table A4).

Technical issues were encountered that affected the manipulation of stress for 7 participants. These participants were therefore not included in the classification analyses.

### 2.6. Machine Learning Models

The self-report and unobtrusive measures were used to predict two types of outcomes: the stress condition to which participants were assigned (*classification*), as discussed in Section 2.6.1, and their performance on a complex synthetic task environment (*regression*), as outlined in Section 2.6.2. During the training of each machine learning model, missing values were imputed using the mean of the corresponding feature from the training set, such that:

(1) $$x_{ij}=\left\{\begin{array}{cc} x_{ij}, & \mathrm{if}\;\mathrm{observed},\\ {\bar{x}}_{i}^{\mathrm{train}}, & \mathrm{otherwise}, \end{array}\right.$$

where ${\bar{x}}_{i}^{\mathrm{train}}$ denotes the mean of feature *i* computed only from the training partition and $x_{ij}$ is the $j^{th}$ sample of feature *i*. The default parameters for the decision tree (classification) and random forest (regression) models as implemented in scikit-learn (version 1.8.0; https://scikit-learn.org/stable/, last accessed on 20 June 2026) were used. These defaults include built-in stopping criteria that prevent overfitting, allowing the assessment of the baseline performance without manual tuning. These models were selected because they are well-established, interpretable machine learning methods that provide a consistent baseline across all modalities, enabling a fair comparison of the criterion-related validity of the different stress measures while minimizing the influence of model complexity on the observed performance. Hyperparameter sensitivity analyses were conducted and indicated that model performance was largely stable across a reasonable range of hyperparameter values (see Tables S1 and S2 of the Supplementary Material).

#### 2.6.1. Classification Models

The objective of classification models in the present study is to predict the stress conditions (i.e., *no-stress*, *low-stress*, and *high-stress*) based on the available measured features. Because the conditions are designed to simulate different levels of stress, a high model performance would suggest that the selected features are related to stress. Three classification analyses were conducted using different combinations of trials and features. That is, the stress condition was predicted (1) first using data from all modalities (i.e., self-report, physiological, computer interactions, and facial) and all experimental trials (Trials 2 to 4), (2) then using data from all experimental trials by modality to compare the performance of different modalities, and (3) finally using data from all modalities by trial to examine the stability of the performance of features over time. The analyses were conducted by fitting decision tree models to the selected features with each trial serving as a sample. All models were trained using a leave-one-participant-out cross-validation protocol to ensure robustness and generalizability of the results. The macro-F1 score for each analysis was used to evaluate the classification performance with a chance macro-F1 score at 33% for this three-way classification task: $\mathrm{Macro}\text{-}F1=\frac{1}{C}\sum_{i=1}^{C}F1_{i}$, where *C* is the total number of classes and $F1_{i}$ is the F1-score for class *i*. Additionally, per-class F1 scores with all features were presented to illustrate class-specific performance. To determine whether the classification performance exceeded chance level, two-sample *t*-tests were conducted by comparing the macro-F1 scores obtained across 25 distinct LOSO runs against the chance level of 0.3333.

#### 2.6.2. Regression Models

The objective of the regression models is to predict participants’ score on the performance task (*Crisis*). Scores in the present data range from 0 to 287.5 after adjusting for the length of the trial, as indicated in the Section 2.2. Regression analyses were conducted using different combinations of trials and features similar to those in the classification analyses. The only difference is that Trial 1 was not excluded in any of the analyses as a relationship is expected between *Crisis* performance and measures of stress even when stress is not manipulated. A random forest regressor was trained using a leave-one-participant-out cross-validation approach to avoid overfitting. Pearson’s correlation was chosen as the performance metric as it effectively captures trends in performance rather than absolute score values, making it suitable given the large score range, and was computed as:

(2) $$r=\frac{\sum_{j=1}^{n}\left(z_{j}-\bar{z}\right)\left(y_{j}-\bar{y}\right)}{\sqrt{\sum_{j=1}^{n}{\left(z_{j}-\bar{z}\right)}^{2}}\sqrt{\sum_{j=1}^{n}{\left(y_{j}-\bar{y}\right)}^{2}}}$$

where $z_{j}$ and $y_{j}$ are the actual and predicted outcome values, respectively, for sample *j*, $\bar{z}$ and $\bar{y}$ are the corresponding means, and *n* is the total number of samples. Correlations are a standardized statistic that do not depend on the scale of the outcome variable and therefore allow for comparisons with the results of other studies that examine the topic of stress measurement within and/or across disciplines (e.g., computer science and psychology). Statistical significance was assessed by first estimating a chance Pearson’s correlation, computed as the correlation between the true outcome labels and a constant predictor equal to the mean outcome value. Two-sample *t*-tests were then performed to compare the Pearson’s correlation coefficients obtained across 25 distinct LOSO runs against this chance correlation. In addition, two-sample *t*-tests were conducted to compare the Pearson correlation coefficients obtained from the 25 independent LOSO runs between the models that contain all modalities and each individual modality.

## 3. Results

### 3.1. Classification Models Results

**Figure 3:**
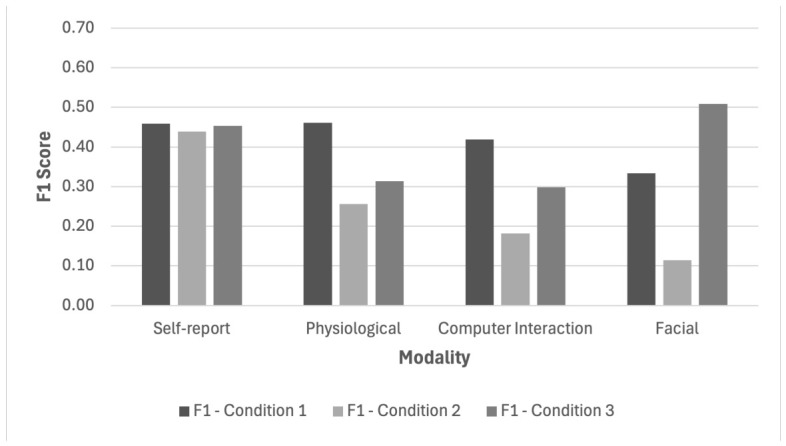
F1 Scores for classifying the stress condition (i.e., *No-stress*, *Low-stress*, and *High-stress*) by modality.

**Figure 4:**
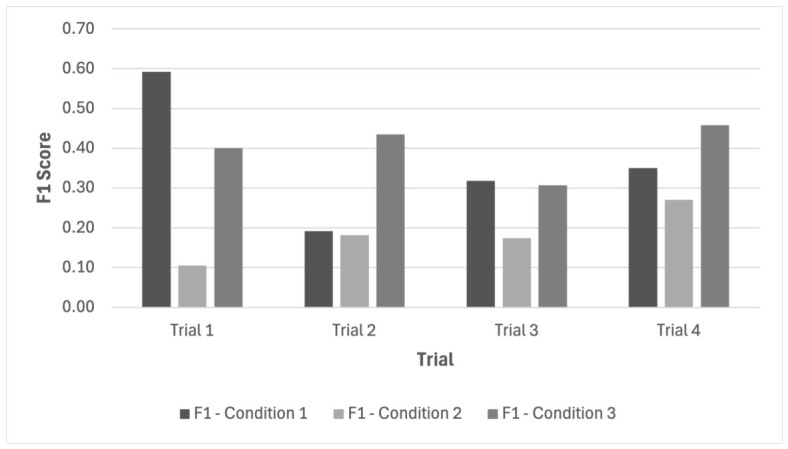
F1 Scores for classifying the stress condition (i.e., *No-stress*, *Low-stress*, and *High-stress*) by trial.

The macro-F1 scores for each classification analysis (i.e., *All Modalities and All Experimental Trials*, *All Experimental Trials by Modality*, *All Modalities by Trial*) are reported in Table 3, and the per-class F1 scores by modality and trial are depicted in Figure 3 and Figure 4, respectively. Due to the relatively high-dimensional feature space compared to the sample size, additional classification experiments using feature selection were conducted, in which the resulting classification performance remained comparable to that obtained using the full feature set (Table A5).

#### 3.1.1. All Modalities and All Experimental Trials

Model performance was first investigated using all available features and experimental trials. Specifically, the analysis used data from all modalities but was limited to Trials 2 to 4 (experimental trials) because no stress manipulation was implemented during Trial 1 across the conditions. The feature set included 31 physiological features, 10 mouse interaction features, 205 facial action units, and three self-report composite scores per trial, collected over three trials. This resulted in a total of 747 features (249 per trial × 3 trials). The macro-F1 score obtained using this complete feature set is 0.4153, which is significantly higher than chance level (t[24]=12.48, p<0.01). This suggests that the full feature set contains information relevant to stress levels.

#### 3.1.2. All Experimental Trials by Modality

The classification by modality across experimental trials (i.e., excluding Trial 1) was then conducted to determine which modality is most effective in identifying the stress condition. Classification using self-report measures achieved a macro-F1 score of 0.4501 significantly outperforming chance (t[24]=22.75, p<0.01). Physiological features yielded a macro-F1 score of 0.3439, but did not differ significantly from chance (t[24]=−0.17, p=0.864). Finally, the analysis using computer interaction features resulted in a score of 0.2997, while that using facial action units features yielded a score of 0.3187. Therefore, self-report measures contribute the most to stress-level identification and the other three modalities’ macro-F1 scores were around chance level (0.3333). In examining the results across stress conditions, self-reported measures did not show notable variation. However, the *no-stress* and *high-stress* conditions yielded overall higher macro-F1 scores compared to the *low-stress* condition for the unobtrusive sensor measures, including physiological, computer interaction, and facial features. This pattern may suggest that both the absence of stress and the presence of high stress might have a more pronounced impact on the unobtrusive sensor data, whereas differences in features in the low-stress condition compared to the other conditions may be less easily detectable.

#### 3.1.3. All Modalities by Trial

Finally, due to practice, participants may have habituated to the study protocol over time, leading to different experienced and perceived stress levels during the subsequent trials and potentially affecting the performance of features. To examine this effect, model performance in predicting the stress conditions was evaluated separately for each trial. All available features, including physiological, computer interaction, facial action units, and self-report measures, were concatenated within each trial. The macro-F1 scores for individual trials range from below to slightly above chance level: 0.3660 for Trial 1, 0.2696 for Trial 2, 0.2666 for Trial 3, and 0.3598 for Trial 4. An examination of the results per condition (Figure 4) indicates that the *no-stress* and *high-stress* conditions have overall higher macro-F1-scores compared to the *low-stress* condition. Therefore, these results suggest that the criterion-related validity of features varies over time, potentially due to participants’ adaptation to stress across trials, and it is easier to classify participants into the condition with no manipulation or to the conditions with the strongest manipulation.

#### 3.1.4. Summary of Classification Results

The classification results indicate that the different measures of stress as a whole display some criterion-related validity when operationalized as correctly classifying participants into the stress condition to which they were assigned (macro-F1 score of 0.4153). However, validity levels seem to vary by modality, whereby self-report measures emerged as the strongest modality (macro-F1 score of 0.4501), and by trial, whereby validity was highest using Trial 1 (macro-F1 score of 0.3660).

### 3.2. Regression Models Results

**Figure 5:**
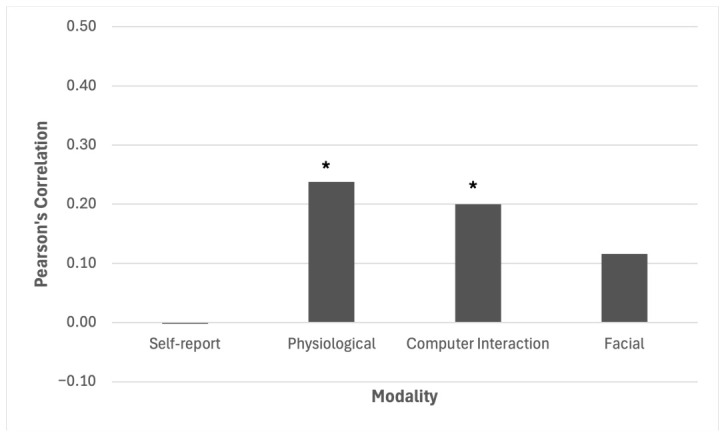
Pearson’s correlations for predicting task performance by modality. Nothing is displayed for the self-report modality because its correlation is −0.002, which is functionally zero. * *p* ≤0.001.

**Figure 6:**
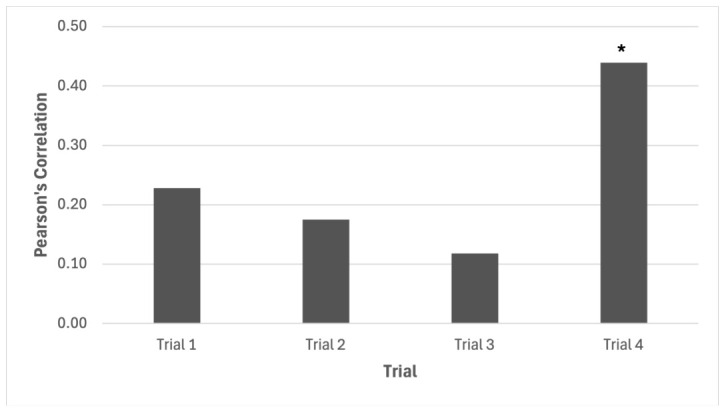
Pearson’s correlations for predicting task performance by trial. * *p* ≤0.001.

Results of the different regression analyses that predict participants’ scores on the performance task (*Crisis*) (i.e., *All Modalities and All Trials*, *All Trials by Modality*, *All Modalities per Trial*) are summarized in Table 4. The results by modality and trial are illustrated in Figure 5 and Figure 6, respectively. Similar to the classification analyses, applying feature selection yielded regression performance that was comparable to the baseline models across most analyses (Table A6).

#### 3.2.1. All Modalities and All Trials

Task (*Crisis*) performance was first predicted using all available features from Trial 1 to Trial 4. This model achieved an r(263)=0.313, *p* < 0.001, indicating a statistically significant relationship between all features and task performance. Furthermore, this correlation was significantly greater than the chance Pearson’s correlation (t[24]=120.112, p<0.01).

#### 3.2.2. All Trials by Modality

The predictive power of different modalities in estimating participants’ task performance was also examined using data from all trials (Trial 1 to Trial 4). When using only self-reported measures as features, the model achieved an r(263)=−0.002, p=0.970, indicating no meaningful relationship. Physiological measures yielded an r(263)=0.238, p<0.001, which was significantly greater than the chance Pearson’s correlation (t[24]=85.533, p<0.01). Mouse interaction features achieved an r(263)=0.200, p=0.001, significantly greater than the chance Pearson’s correlation (t[24]=18.484, p<0.01). Facial action unit features resulted in an r(263)=0.116, p=0.059, significantly greater than the chance Pearson’s correlation (t[24]=25.872, p<0.01). Therefore, across modalities, physiological features performed best. Interestingly, self-reported measures, which were previously effective in predicting stress levels (stress conditions), offered little value in task performance prediction, suggesting that self-reported stress levels and task performance might not be meaningfully related. The model with all modalities achieved significantly higher Pearson correlation coefficients than the individual modalities. For instance, the Pearson’s correlation obtained using all modalities was significantly greater than that obtained using the physiological modality (t[24]=18.24, p<0.001).

#### 3.2.3. All Modalities by Trial

Model performance was also examined separately for each trial, given potential practice effects as indicated by the increase in task performance over trials (see Table 2). Using only Trial 1 data, the model achieved an r(65)=0.228, p=0.064. For Trial 2, the correlation was r(63)=0.175, p=0.164, while Trial 3 yielded the lowest correlation, r(66)=0.118, p=0.339. In contrast, Trial 4 showed the highest correlation, r(63)=0.439, p<0.001. Similar to the classification analyses, the results suggest that the predictive power of features varies over time.

#### 3.2.4. Summary of Regression Results

Similar to the classification results, the regression results indicate that the different measures of stress as a whole have some criterion-related validity when operationalized in terms of their relationship to task performance (r=0.313). However, validity levels appear to vary by modality, whereby physiological features emerged as the strongest modality (r=0.238), and by trial, whereby validity was highest in Trial 4 (r=0.439).

### 3.3. Feature Selection Analysis

To provide greater insight into the features contributing to the prediction models, we examined the consensus features that were consistently selected across leave-one-participant-out cross-validation folds (Table 5). The selected physiological features reflect changes in autonomic nervous system regulation and have been widely associated with acute stress through shifts in sympathetic and parasympathetic activity. The consistent selection of physiological features across cross-validation folds indicates that variations in sympathetic and parasympathetic activity provide valuable information for discriminating between stress levels and estimating task performance. Mouse interaction features may reflect changes in motor behavior. Therefore, they provided informative interaction patterns to discriminate stress levels. Notably, however, these motor changes were not associated with task performance. Finally, several facial action units were consistently identified suggesting that subtle changes in facial muscle activation may be associated with increased cognitive effort and tension, which are useful for discriminating stress conditions and predicting task performance.

## 4. Discussion

The study sought to examine the extent to which unobtrusive measures of stress can serve as valid substitutes for self-report measures. To achieve this objective, the study used features from four different modalities (i.e., self-report, physiological, computer interaction, and facial) to predict two outcomes (the stress condition and task performance) using data from multiple time points (i.e., 4 trials).

Based on the results, a number of summary statements and discussion points can be noted. First, the analyses *by modality* indicated that the stress condition was predicted by the self-report measures (macro−F1=0.45), whereas task performance was predicted by physiological (r=0.24) and mouse interaction (r=0.20) measures. The observed relationship between task performance and physiological and mouse interaction measures suggests that the criterion-related validity of these two modalities is not limited to brief controlled tasks (e.g., Stroop Color-Word Test), which are commonly examined in the literature but also extend to performance on more complex tasks. Pertaining to the stress condition, the finding that this outcome was predicted exclusively by self-report measures suggests that in spite of their recognized limitations as reviewed in the introduction, self-report measures can in some instances have higher criterion-related validity than other modalities. Taken together, the different results of the stress condition and task performance also indicate that the validity and utility of modalities depend on the outcome of interest. One should note that the two outcomes examined in the present study are qualitatively different, which may explain why they were predicted by different modalities. The stress condition is a variable that was manipulated to introduce meaningful variance in the level of stress experienced by participants. On the other hand, performance on complex tasks such as that used in the present study is the result of cognitive, emotional, and behavioral processes that participants engaged in, and this performance could potentially be influenced by the levels of stress they experienced.

One may also speculate about possible reasons for the absence of relationships between specific modalities and outcomes. For instance, the finding that physiological measures did not predict the stress condition is at odds with prior studies that reported an association between physiological measures and stress [22]. This null result may, however, be due to the type of task used in the present study. Indeed, much of the prior work on stress manipulation has relied on controlled tasks such as mental arithmetic tests and the Stroop Color-Word Test, which are significantly shorter in duration and more specialized in specific skills or abilities compared to *Crisis*, which is relatively longer (6 to 10 min), open-looped, and involves various psychological processes. Another surprising result worth noting is that self-report measures did not predict task performance. Prior work suggests that weak correlations between self-report and behavioral (or performance-based) measures may be due to the poor reliability of behavioral measures [45]. However, this explanation is unlikely to be the case in the present study as *Crisis* performance displayed high test-retest reliability estimates (i.e., >0.65; see Section 2). Thus, a more plausible explanation for the absence of a relationship between self-report and task performance in this instance is likely the limited variability in *Crisis* performance scores resulting from the difficulty of the task. As reflected in Table 2, the maximum possible score is 600, whereas the *mean* task performance scores across trials ranged from 4.32 to 136.97.

Second, the analyses of *all modalities by trial* indicated that model predictions of the outcomes varied from one trial to another. Specifically, for task performance, the best predictions were obtained in Trial 1 (r=0.23) and Trial 4 (r=0.44). Similarly for the stress condition, among the trials where stress was manipulated (i.e., Trial 2 to 4), classification was best in Trial 4 (macro−F1=0.36). These findings could be related to the unique experiences of participants in these two trials. That is, Trial 1 is different from other trials in that the design of the study and the experience of the task are still novel to the participants, and therefore, this trial requires more cognitive processing. Trial 4 is also unique in that it is the last trial of the task. Specifically, by Trial 4, participants might have had sufficient practice to get better at the task. In addition, being aware that Trial 4 is the last one, participants may also have exerted all their efforts in this trial to maximize their chances of receiving the additional performance-based compensation. More generally, the variability in the results by trial indicates that the criterion-related validity of stress features is trial-dependent with potentially different psychological underpinnings at different time points.

Third, the analyses of *all modalities and trials* predicted both the stress condition (macro−F1=0.42), and task performance (r=0.31). This suggests that features from different modalities and trials as a whole contain information relevant to stress, which is consonant with the previously discussed results of the analyses by modality and trial. However, interestingly, the predictions using all modalities and trials were not the best across all the analyses conducted. Specifically, the model with all modalities and trials (macro−F1=0.42) predicted the stress condition worse than both the model with only self-report measures (macro−F1=0.45) and the model with only Trial 1 (macro−F1=0.37). Similarly, pertaining to task performance, the model with all modalities and trials (r=0.31) predicted this outcome worse than the model with only Trial 4 (r=0.45). These results suggest that more modalities are not necessarily better and may instead introduce noise to the predictions. Practically, this also means that practitioners may not need to measure stress using all available modalities and time windows to maximize predictions of the outcomes of interest. Another possible explanation is that all modalities contribute to a high-dimensional feature space, which may be challenging for the models to learn effectively given the limited number of data samples relative to the number of features. A more targeted and parsimonious approach may be more advantageous, especially given the logistical challenges and costs of data collection.

### Limitations and Suggestions for Future Research

Some limitations of the present study serve as the basis for future research. The use of a sample of college students who performed a single synthetic complex task in a protocol that lasted approximately 2.5 h raises potential generalizability concerns. Future research should seek to replicate the findings reported in other settings. To better assess the predictive power of stress modalities, future work may also consider using a stronger manipulation of stress. The same types of stress demands as those used in the present study (i.e., time constraints and distracting audio) could be manipulated with stronger contrasts. Alternatively, one can also introduce other types of demands to the task environment such as lighting [46] or temperature [47].

Furthermore, prior work has indicated that stress is a multivariate construct [28]. Therefore, although the present study examined multiple modalities of stress (i.e., self-report, physiological, computer interaction, and facial), additional features from other modalities such as oculometry and speech could also be considered. And because such features can be unobtrusively collected from wearable sensors, microphones, and cameras, their inclusion alongside self-report measures would allow for a more comprehensive examination of the extent to which unobtrusive measures of stress can serve as valid substitutes for self-report measures.

Third, the finding that model predictions of the outcomes in the present study varied by trial calls for further research to identify the optimal time and the frequency of measurement that maximize predictions. Relatedly, because predictions of the outcomes also varied by modality, another pertinent research question is whether predictions are influenced by an interaction of both the time and the modality of measurement.

Finally, the present study employed relatively simple multimodal fusion approaches to establish a benchmark for evaluating the ability of multiple modalities to predict stress-related outcomes using the collected data. This design enabled differences in predictive performance to be more directly attributed to the measurement modalities rather than to variations in model complexity. Advanced multimodal fusion approaches, hybrid/fuzzy classification techniques, and probabilistic approaches to model uncertainty in the various multimodal measurements should be explored as future work to further improve predictive performance while preserving the relative strengths of the different stress measurement modalities observed in this study.

## 5. Conclusions

This study examined whether unobtrusive measures of stress can serve as valid substitutes for self-report measures. The study used self-reported, physiological, computer interaction, and facial features to predict the stress condition and task performance using data collected from four trials in a synthetic task environment that simulates responding to a disaster. Overall, the results indicated that whereas the unobtrusive measures individually predicted task performance better than self-report measures, self-report measures predicted the stress condition better than the unobtrusive measures. Thus, unobtrusive measures do not always predict outcomes better than self-report measures in spite of their limitations, and the criterion-related validity and utility of modalities may depend on the outcome of interest.

## Figures and Tables

**Table 1:** Sample demographic information.

Variable	Classification	*n*	%
Sex	Male	46	58.97
	Female	32	41.03
Ethnicity	Asian	34	43.59
	White	25	32.05
	Hispanic	13	16.67
	Two or More Races	3	3.85
	Black	2	2.56
	Pacific Islander	1	1.28
Degree Level	Undergraduate	49	62.82
	Graduate	28	35.90
	Post-Graduate	1	1.28
Employment Status	Not employed	49	62.82
	Employed Part-Time	23	29.49
	Employed Full-Time	6	7.69

**Table 2:** Descriptive statistics for the self-report and performance variables.

Category	Variable	Measure	*M*	*SD*
Self-report measures of stress	State anxiety	STAI Form Y-1		
	Post-Trial 1		2.37	0.59
	Post-Trial 2		2.35	0.59
	Post-Trial 3		2.26	0.57
	Post-Trial 4		2.12	0.52
	Momentary stress	Single item of momentary stress		
	During Trial 1		5.23	0.92
	During Trial 2		5.24	1.12
	During Trial 3		5.13	1.15
	During Trial 4		4.74	1.38
	Task load	NASA-TLX		
	Post-Trial 1		64.89	18.43
	Post-Trial 2		70.70	14.85
	Post-Trial 3		69.35	16.22
	Post-Trial 4		67.63	17.86
Performance	Performance Trial 1	*Crisis in the Kodiak:*	4.32	21.20
	Performance Trial 2	*Oilrig Search and Rescue*	54.19	42.34
	Performance Trial 3		93.86	64.60
	Performance Trial 4		136.97	56.03

**Table 3:** F1 Scores for classifying the stress condition (i.e., *No-stress*, *Low-stress*, and *High-stress*) using different modalities and trials.

Analysis	Macro F1	F1	F1	F1
		Condition 1	Condition 2	Condition 3
		No Stress	Low Stress	High Stress
*All Modalities and All Experimental Trials*	0.4153	0.4590	0.3684	0.4186
*All Experimental Trials by Modality*				
Self-report	0.4501	0.4583	0.4390	0.4528
Physiological	0.3439	0.4615	0.2564	0.3137
Comp. Interact.	0.2997	0.4194	0.1818	0.2979
Facial	0.3187	0.3333	0.1143	0.5085
*All Modalities by Trial*				
Trial 1	0.3660	0.5926	0.1053	0.4000
Trial 2	0.2696	0.1923	0.1818	0.4348
Trial 3	0.2666	0.3182	0.1739	0.3077
Trial 4	0.3598	0.3509	0.2703	0.4583

Comp. Interact.: Computer Interaction.

**Table 4:** Pearson’s correlations for predicting task performance using different modalities and trials.

Analysis	Pearson’s Correlation	*p*-Value
*All Modalities and All Trials*	0.310	<0.001
*All Trials by Modality*		
Self-report Measures	−0.002	0.970
Physiological	0.238	<0.001
Computer Interaction	0.200	0.001
Facial Action Units	0.116	0.059
*All Modalities by Trial*		
Trial 1	0.228	0.064
Trial 2	0.175	0.164
Trial 3	0.118	0.339
Trial 4	0.439	<0.001

**Table 5:** Features selected consistently across leave-one-participant-out cross-validation folds for the stress condition classification and the task performance regression analyses conducted across all modalities and all trials.

Analysis	Selected Features
*Stress Condition*	
Physiological	NNI_mean, NNI_sd, NNI_20, NNI_50, p_NNI_20, p_NNI_50, LF_HF_ratio
Computer Interaction	X-axis.max, Clicks
Facial Action Units	AU 6 (standard deviation), AU 15 (mean), AU 18 (mean), AU 6 (mean)
*Task performance*	
Physiological	LF_HF_ratio, LFnu, HFnu, SCL, SCRAmp, SCRFreq, ACC_enrg, BVP_mean, HR_mean, IBI, NNI_20, pNNI_20, VLF
Facial action units	AU 39 (mean), AU 4 (standard deviation), AU 14 (standard deviation), AU 12 (standard deviation)

## Data Availability

Consonant with the approved IRB protocol, the data presented in this study can be requested using this link: https://sites.google.com/colorado.edu/sums-dataset/home (accessed on 8 May 2026).

## Supplementary Materials

The following supporting information can be downloaded at: https://www.mdpi.com/article/10.3390/s26154962/s1, Table S1: Sensitivity analysis of the decision tree classifier with respect to the maximum tree depth (*max_depth*) and the minimum number of samples required to be at a leaf node (*min_samples_leaf*) hyperparameters. Macro-F1 scores are reported for the model that contains all modalities and each individual modality; Table S2: Sensitivity analysis of the random forest regression model with respect to the number of estimators (*n_estimators)*, maximum tree depth (*max_depth*), and the number of features considered at each split (*max_features*) hyperparameters. Pearson’s correlations are reported for the model that contains all modalities and each individual modality.

## Appendix A

**Table A1:** List and description of physiological measures of stress.

Indicator	Description	Device
SCL	Skin conductance level [EDA]	Empatica E4
SCRAmp	Skin conductance response amplitude [EDA]	Empatica E4
SCRFreq	Skin conductance response frequency [EDA]	Empatica E4
IBI	Interbeat interval [BVP]	Empatica E4
HR_mean.1	Mean heart rate [BVP]	Empatica E4
BVP_mean	Mean BVP [BVP]	Empatica E4
ACC_enrg	Energy of acceleration signal [ACC]	Empatica E4
NNI_mean	Mean of N-N intervals [ECG]	Actiwave
NNI_sd	Standard deviation of N-N intervals [ECG]	Actiwave
NNI_range	Range of N-N intervals [ECG]	Actiwave
NNI_median	Median of N-N intervals [ECG]	Actiwave
NNI_cv	Coefficient of variation of N-N intervals [ECG]	Actiwave
NNI_50	Number of successive NNI pairs differing by >50 ms [ECG]	Actiwave
NNI_20	Number of successive NNI pairs differing by >20 ms [ECG]	Actiwave
p_NNI_50	Percentage of successive NNI pairs differing by >50 ms [ECG]	Actiwave
p_NNI_20	Percentage of successive NNI pairs differing by >20 ms [ECG]	Actiwave
NNIDiff_sd	Standard deviation of successive differences between NNIs [ECG]	Actiwave
NNIDiff_rms	Root mean square of successive differences between NNIs [ECG]	Actiwave
NNIDiff_cv	Coefficient of variation of successive differences between NNIs [ECG]	Actiwave
HR_mean.2	Mean heart rate [ECG]	Actiwave
HR_max	Max heart rate [ECG]	Actiwave
HR_min	Min heart rate [ECG]	Actiwave
HR_sd	Standard deviation of heart rate [ECG]	Actiwave
VLF	Very low-frequency power [ECG]	Actiwave
LF	Low-frequency power [ECG]	Actiwave
HF	High-frequency power [ECG]	Actiwave
LFnu	Normalized low-frequency power [ECG]	Actiwave
HFnu	Normalized high-frequency power [ECG]	Actiwave
LF_HF_ratio	Ratio of low-frequency to high-frequency power [ECG]	Actiwave
Total_power	Total spectral power [ECG]	Actiwave
HR_mean.3	Mean heart rate [HR]	Actiwave

**Table A2:** List and description of computer interaction measures of stress.

Indicator	Description
Clicks	Number of mouse clicks
X-axis.min	Minimum coordinate of clicks on X-axis
X-axis.max	Maximum coordinate of clicks on X-axis
Y-axis.min	Minimum coordinate of clicks on Y-axis
Y-axis.max	Maximum coordinate of clicks on Y-axis
InitiationTime	Time to first click
ClickInterval.Speed	Average time between clicks
ClickInterval.Acc	Average difference in time between clicks
Reversals X-axis	Number of changes in clicks along the X-axis
Reversals Y-axis	Number of changes in clicks along the Y-axis

**Table A3:** List and description of facial measures of stress.

Indicator	Description
AU 1	Inner brow raiser
AU 2	Outer brow raiser
AU 4	Brow lowerer
AU 5	Upper lid raiser
AU 6	Cheek raiser
AU 7	Lid tightener
AU 9	Nose wrinkler
AU 10	Upper lip raiser
AU 11	Nasolabial deepener
AU 12	Lip corner puller
AU 13	Sharp lip puller
AU 14	Dimpler
AU 15	Lip corner depressor
AU 16	Lower lip depressor
AU 17	Chin raiser/Lip pucker
AU 18	Lower lip depressor
AU 19	Tongue show
AU 20	Lip stretcher
AU 22	Lip funneler
AU 23	Lip tightener
AU 24	Lip pressor
AU 25	Lips part
AU 26	Jaw drop
AU 27	Mouth stretch
AU 32	Lip bite
AU 38	Nostril dilator
AU 39	Nostril compressor
AUL 1	Left inner brow raiser
AUR 1	Right inner brow raiser
AUL 2	Left outer brow raiser
AUR 2	Right outer brow raiser
AUL 4	Left brow lowerer
AUR 4	Right brow lowerer
AUL 6	Left cheek raiser
AUR 6	Right cheek raiser
AUL 10	Left upper lip raiser
AUR 10	Right upper lip raiser
AUL 12	Left nasolabial deepener
AUR 12	Right nasolabial deepener
AUL 14	Left dimpler
AUR 14	Right dimpler

## Appendix B

**Table A4:** Study procedure.

Scheduled Activity	Activity Length
**Informed consent**	5 min
**Hearing ability test:** All participants passed the test and were thus eligible to proceed with their participation in the study. Furthermore, no significant difference was found between the three stress conditions (Fs<3.02, ps>0.05), indicating that the random assignment was effective.	5 min
**Equipping Participants with Physiological Devices** (i.e., Actiwave Cardio and Empatica E4)	10 min
**Relaxation Video:** Consisted of guided mindfulness meditation accompanied by calming music and camera shorts of sunlit forests [48,49,50] meant to engender a physiological baseline	5 min
**Spoken Overview of** ***Crisis***	2 min
**Trial 1**	
Performance T1	10 min
Momentary stress T1	45 s
State anxiety T1	3 min
Task load T1	2 min
***Crisis*** **Tutorial:** a detailed, self-paced tutorial of *Crisis*	15 min
**Trial 2**	
Performance T2	6, 8, or 10 min ^a^
Momentary stress T2	45 s
State anxiety T2	3 min
Task load T2	2 min
**Trial 3**	
Performance T3	6, 8, or 10 min ^a^
Momentary stress T3	45 s
State anxiety T3	3 min
Task load T3	2 min
**Trial 4**	
Performance T4	6, 8, or 10 min ^a^
Momentary stress T4	45 s
State anxiety T4	3 min
Task load T4	2 min
**Wrapping Up**	
Demographics	5 min
Remove physiological devices	5 min
Debriefing	2 min

^a^ The duration of Trials 2, 3 and 4 varied by stress condition: 10 min (*no stress*), 8 min (*low stress*), and 6 min (*high stress*). Although the whole research protocol lasted 2.5 h, activities for the data used in the present study lasted 117, 111, and 105 min for the three conditions, respectively. T = Trial.

## Appendix C

**Table A5:** Comparison of F1 scores for classifying the stress condition (i.e., *No Stress*, *Low Stress*, and *High Stress*) with and without feature selection using different modalities and trials.

Analysis	Without Feature	With Feature
	Selection	Selection
*All Modalities and All Experimental Trials*	0.415	0.386
*All Experimental Trials by Modality*		
Self-report	0.450	0.450
Physiological	0.356	0.336
Computer Interaction	0.300	0.320
Facial	0.319	0.304
*All Modalities by Trial*		
Trial 1	0.366	0.398
Trial 2	0.270	0.252
Trial 3	0.252	0.253
Trial 4	0.360	0.363

**Table A6:** Comparison of Pearson’s correlations for predicting task performance with and without feature selection using different modalities and trials.

Analysis	Without Feature	With Feature
	Selection	Selection
*All Modalities and All Experimental Trials*	0.311 *	0.356 *
*All Experimental Trials by Modality*		
Self-report	−0.002	−0.002
Physiological	0.238 *	0.238 *
Computer Interaction	0.200	0.200
Facial	0.116	0.116
*All Modalities by Trial*		
Trial 1	0.228	0.248
Trial 2	0.175	0.050
Trial 3	0.118	0.167
Trial 4	0.439 *	0.465 *

* p<0.05.
