# Supplementary material for: A Comparison of Self-Reports and Unobtrusive Multimodal Measures of Stress in a Realistic Simulated Task Environment
Source: Sensors (Basel). 2026 Aug 5;26(15):4962. doi: 10.3390/s26154962 (PMC13469585; doi:10.3390/s26154962)
Supplement: Supplementary file 1 [file sensors-26-04962-s001.zip › sensors-4336493-supplementary.pdf]

Supplementary Material

Table S1: Sensitivity analysis of the decision tree classifier with respect to the maximum tree depth (*max_depth*) and the minimum number of samples required to be at a leaf node (*min_samples_leaf*) hyperparameters. Macro-F1 scores are reported for the model that contains all modalities and each individual modality.

**Hyperparameter**	**All**	**Self-report**	**Physiological**	**Computer Interaction**	**Facial**
max_depth					
3	0.4690	0.3268	0.3219	0.3045	0.2600
5	0.3499	0.4347	0.3349	0.2374	0.3171
10	0.4153	0.4501	0.3557	0.2697	0.3187
None	0.4153	0.4501	0.3439	0.2997	0.3187
min_samples_leaf					
1	0.4153	0.4501	0.3557	0.2997	0.3187
5	0.4543	0.5245	0.5382	0.3032	0.3077
10	0.2640	0.3300	0.4266	0.5079	0.3321

Table S2: Sensitivity analysis of the random forest regression model with respect to the number of estimators (*n_estimators)*, maximum tree depth (*max_depth*), and the number of features considered at each split (*max_features*) hyperparameters. Pearson's correlations are reported for the model that contains all modalities and each individual modality.

**Hyperparameter**	**All**	**Self-report**	**Physiological**	**Computer Interaction**	**Facial**
max_depth					
50	.3218	−.0151	.1853	.1912	.1180
100	.3110	−.0028	.2368	.2000	.1109
200	.3069	−.0171	.2345	.2140	.1118
min_samples_leaf					
5	.2988	.0511	.2345	.2299	.1123
10	.3098	−.0056	.2324	.2008	.1051
None	.3110	−.0028	.2368	.2000	.1109
max_features					
0.3	.3318	−.0126	.2659	.2323	.1529
0.5	.3113	−.0126	.2416	.2261	.0978
1.0	.3110	−.0028	.2368	.2000	.1109
